# The Rates of Cesarean Section Deliveries According to Robson Classification System During the Year of 2018 Among Patients in King Abdul-Aziz Medical City, Jeddah, Saudi Arabia

**DOI:** 10.7759/cureus.11529

**Published:** 2020-11-17

**Authors:** Shaymaa M Alsulami, Mohammed T Ashmawi, Rafeef O Jarwan, Israa A Malli, Suheal K Albar, Hatim M Al-Jifree

**Affiliations:** 1 Medicine, King Saud Bin Abdulaziz University for Health Sciences, Jeddah, SAU; 2 Obstetrics and Gynaecology, King Abdul-Aziz Medical City, Jeddah, SAU; 3 Basic Medical Sciences, King Saud Bin Abdulaziz University for Health Sciences, Jeddah, SAU; 4 Medicine, Ibn Sina National College, Jeddah, SAU; 5 Gynecologic Oncology, King Abdul-Aziz Medical City, Jeddah, SAU

**Keywords:** tgcs, robson, cesarean section, cesarean birth, cesarean delivery, svd.

## Abstract

Background: The rate of cesarean section (CS) births has been rapidly increasing in Saudi Arabia during the last two decades. Using the Robson Ten Group Classification System (TGCS) to classify and analyze the causes of the high CS rate.

Objective: To assess the increasing rates of CS by the implementation of the Robson TGCS on all CS births in our chosen population.

Study design: An observational, cross-sectional study conducted among all deliveries at the King Abdul-Aziz Medical City (KAMC), Jeddah, Saudi Arabia during most of 2018. Over the study period, 3168 births were enrolled in the study.

Results: The analysis of 3168 births, where 870 women gave birth through CS, resulted in a CS rate of 27.5%. The three major TGCS which contributed to the CS rate were group 5, 2 (divided into 2A and 2B), and 3. Class 5 (Previous CS, single cephalic, ≥37 weeks) contributed the most to the CS rate by 36.5%. Followed by Class 2 (divided into 2A; nulliparous, singleton, cephalic, ≥37 weeks, induced labor and 2B; nulliparous, singleton, ≥37, pre-labor CS) which contributed by 12.9%. Class 3 (multiparous (no previous CS), singleton, ≥37 weeks, spontaneous labor) was the third-highest contributing group by 9.2%. Women who gave birth spontaneously and vaginally were 1403 (44.3%) where women whose labor was induced were 1286 (40.6%).

Conclusion: The CS rate in KAMC was 27.5%. After classifying these patients according to the TGCS, Class 5 had the largest percentage of patients going for CS (36.2%). While they are individually low together, Robson classes from Class 1 to 4 (which are considered as low-risk classes) were responsible for 37.8% of the patients going for CS. Since the previously mentioned groups are considered low-risk they should be targeted by health institutions to reduce the CS rate. Improved education of nulliparous and multiparous women who never underwent a CS to prevent nonmedically indicated CS is in order, to preclude repeated CS births in the future and further increase the CS rate.

## Introduction

Cesarean section (CS) is a surgical procedure where the mother’s abdomen and uterus are incised to deliver one fetus or more [1]. A CS is executed when medically justified by certain complications during either pregnancy, labor or both such as delivery complications, placenta accreta or previa, and a history of previous CS deliveries [2]. However, CS has a higher rate of maternal and fetal complications compared to vaginal deliveries [3]. Some of these complications cause a higher risk of abdominal organs injury, various infections, placenta previa, placenta accrete, and neonatal respiratory problems [3].

 The World Health Organization (WHO) stated that the acceptable rate of CSs should range from 10% to 15% [4]. However, the CS rate is rapidly increasing, both globally and locally in the previous two decades, evoking worldwide concerns [5]. CS can effectively avert maternal and perinatal morbidity and mortality rates when medically justified. However, there are no current studies that demonstrate any benefits from CS delivery for women or infants, especially considering their associated short-term and long-term risks [5].

 In accordance to the latest data from 150 countries, 18.6% of all births happened by CS, in a range of 6% in the least developed countries, and a much higher rate of 27.2% in the most developed countries. Based on the trend analysis, the CS births rate from 121 countries has increased gradually from 6.7% to 19.1% between the years 1990 and 2014, representing a 12.4% increase, with an average annual increase of 4.4% [6]. The highest increase was noted in Latin America and the Caribbean (19.4%, from 22.8% to 42.2%), where the least increase was observed in Africa (4.5%, from 2.9% to 7.4%) [6].

 In the Middle East, there are a few available studies which are comparable to this study and discusses the same concerning increase in the rates of CS. A similar study to ours was conducted in the United Arab Emirates (UAE), in 2016 [7]. Their study concluded that the CS rate is 33% in the UAE which is considered to be greater than the average global CS rate [7]. Another study in a university hospital in Egypt, published in 2019, resulted in a CS rate of 55% among all deliveries that year [8]. Furthermore, another study from Palestine was published in 2018 with a CS rate of 22.9% [9]. Locally, there are no comparable studies published with the same topic and aim as this study.

 According to the Saudi Ministry of Health (MOH), in 2006 there were 784,145 surgical procedures performed in both governmental and private hospitals, and about 86,197 of the total 784,145 (11%) surgeries were CS [10]. A 10-year review of cesarean deliveries in Saudi Arabia (SA) has revealed an enormous increase in the CS rate by 80% from 1997 to 2006 [10]. Furthermore, through the previous two decades, a noticeable increase in the CS births rate was observed at KAMC, Riyadh, SA, from 8% to 21% between the years 1993 and 2013 [11]. In 2016, the rate of CS in KAMC, Riyadh, has increased to 27% of all deliveries in the center showing that there is an increase in the rates of CS throughout the year [11].

 In this study, we aimed to assess the crude data of obstetric information that is available, and transform those data into useful information by the application of Robson Classification System which is a global standard classification system that classify women into one of the 10 Robson groups based on different obstetric characteristics (number of fetuses, parity, previous CS, onset of labor, fetal presentation, and gestational age), and eventually categorize each cesarean delivery into one of these 10 groups [12]. In this context, after the categorization of each CS, we are aimed to identify the most contributing Robson groups to the overall rate of CS in KAMC, Jeddah, during 2018.

## Materials and methods

Study design, area, and settings

This was a cross-sectional study. The study was conducted in the department of obstetrics and gynecology at KAMC, Jeddah, and the data have been acquired from the hospital’s medical digital records department. The study has a 5% margin of error, a 95% confidence level, and an estimated outcome response of 50%. Therefore, the estimated sample size is determined to be 341. The sample size was calculated using the Raosoft sample size calculator, (http://www.raosoft.com/samplesize.html). However, we decided to include all pregnant women who have received medical care at KAMC, Jeddah during 2018. The estimated number of patients would be >3000 women. 

Identification of study participants

*Inclusion Criteria* 

All pregnant women who received medical care at KAMC, Jeddah during the year of 2018 will be included in the study as a consecutive sampling to include all the population in the desired period.

*Exclusion Criteria* 

Gestational age less than 22 weeks.

Infant weight less than 500 g during delivery.

Data collection process

The data on all deliveries that occurred in KAMC, Jeddah, between January 1st, 2018, until 31st of December, 2018, was attained from the Best Care System, the hospital’s digital records registry. A data collection sheet form was designed, revised thoroughly, and eventually used to collect obstetrical information on both maternal and fetal characteristics. The maternal characteristics were parity, placentation, onset of labor (whether it was spontaneous, induced or elective CS), gestational age, and any previous history of CS. As for the fetal characteristics, data were collected on the number of fetuses, fetal presentation (whether it was cephalic, breech, or transverse). Upon finishing the process of data collection, we categorized each CS into one of the 10-Robson groups according to its maternal and fetal characteristics. No patient was classified into >1 group.

Data analysis 

As for data analysis, all the data which were collected from the medical digital records were entered into an excel sheet and transferred afterwards into the IBM SPSS Statistics software, version 23.0 (IBM Corp, Armonk, New York, USA), V.3 program for statistical analysis. In this study, descriptive analysis of the chosen population was used, and no statistical testing was needed or performed.

Ethical approval 

Before proceeding, approval was sought and granted from the research committee at King Abdullah International Medical Research Center and the Institutional Review Board. No consent form was needed.

## Results

The number of deliveries that met the inclusion and exclusion criteria in KAMC during 2018 was 3640 deliveries. A sample of 3168 deliveries was used in the study. The obstetrical characteristics are demonstrated in Table 1.

**Table 1:** Obstetrical characteristics of all deliveries. CS, cesarean section; SVD, spontaneous vaginal delivery

Demographics	Frequency	Percentage
Parity		
P0	904	28.5%
P1 or more	2264	71.5%
Previous CS		
0 CS	2380	75.1%
1 CS	475	15%
>1 CS	313	9.9%
Placentation		
Normal	3147	99.3%
Abnormal	21	0.7%
Onset of Labor		
Spontaneous	1403	44.3%
Induced	1286	40.6%
Pre-labor CS	479	15.1%
Number of fetuses		
Singleton	2126	98.7%
Multiple	42	1.3%
Gestational Age		
Term	2896	91.4%
Pre-term	272	8.6%
Fetal Presentation		
Cephalic	2966	93.6%
Breech	188	5.6%
Transverse	4	0.4%
Outcome		
SVD	2131	67.3%
Instrumental	167	5.3%
Elective or pre-labor CS	479	15.1%
Emergency CS	391	12.3%

**Figure 1:**
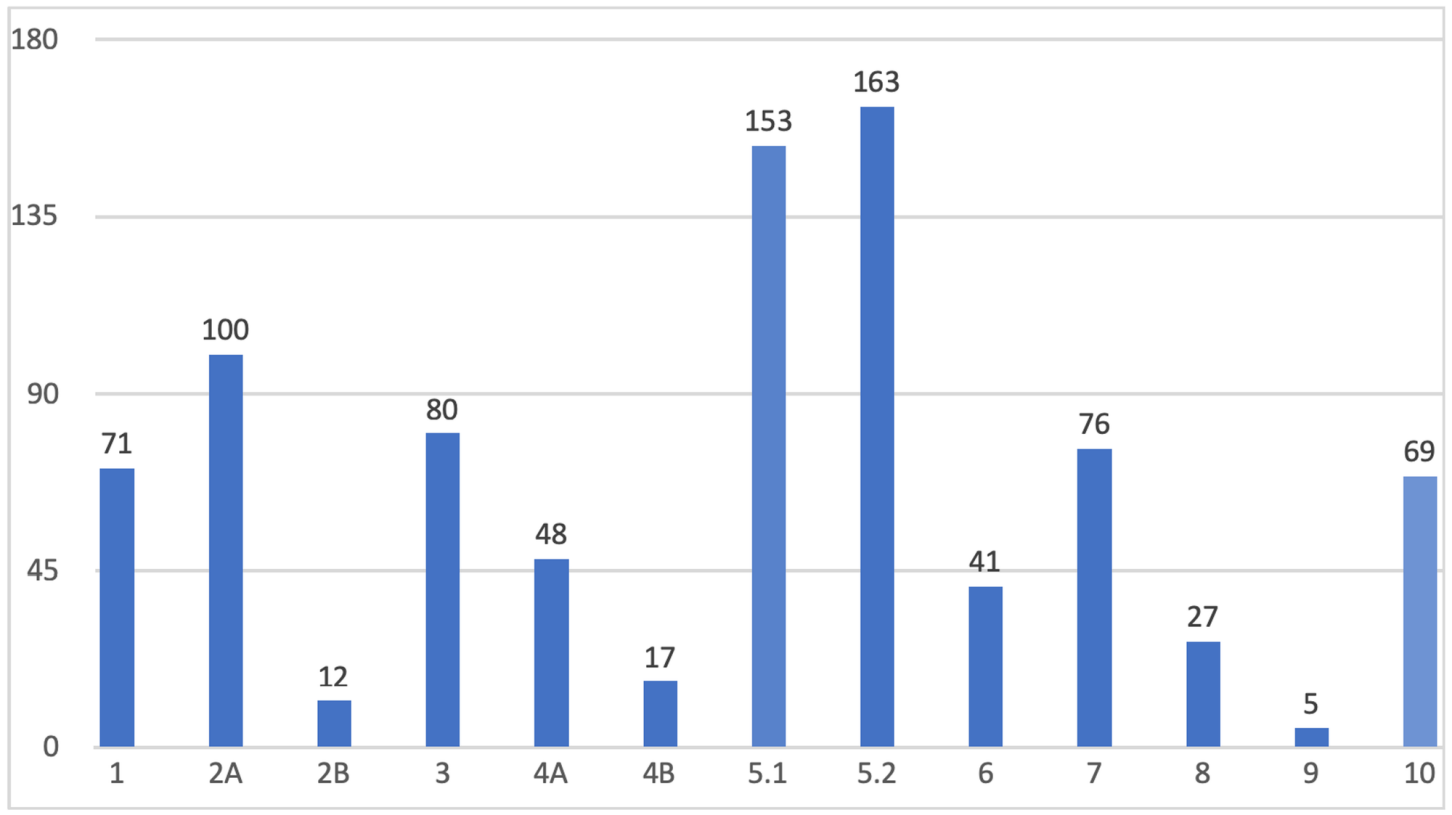
The frequency of cesarean births in each Robson Classification group.

Out of those 3168 women, 904 were nulliparous (28.5%) while 2264 were multiparous women (71.5%). Patients who never had a previous CS were 2380 patients (75.1%) while patients who had only one previous CS were 475 (15%) and patients who had more than one CS were 313 patients (9.9%). Patients with normal placentation were 3147 (99.3%), and patients who presented with placenta previa or accreta were nine patients (0.7%). Patients who had spontaneous labor were 1403 patients (44.3%). Patients whose labor was induced or augmented were 1286 (40.6%), and 479 patients (15.1%) went to CS before labor electively. The patients who were pregnant with a singleton were 3126 patients (98.7%) while 42 patients (1.3%) were multiple pregnancies. Patients who were term pregnancies (≥ 37+0 weeks) were 2896 patients (91.4%) while 272 patients (8.6%) were preterm (≤ 37 weeks). Patients who were cephalic presentation were 2966 patients (93.6%) while 188 patients (5.9%) were breech presentation and 14 patients (0.4%) were transverse. The majority of the patients (2131) delivered vaginally (67.3%) while 870 patients delivered by CS (27.5%), and 167 patients (5.3%) were instrumental deliveries by either ventouse or forceps. The total number of patients who gave birth by CS were 870 patients (27.5%). As shown in Figure 1, of these patients, 71 were classified into Class 1 (8.2%). Class 2 has 113 patients (13%) and was further divided into Class 2A which has 101 patients (11.6%) and Class 2B which has 12 patients (1.4%). Class 3 has 80 patients (9.2%). Furthermore, 65 patients are classified into Class 4 (7.5%) and were further divided into 4A 48 patients (5.5%) and 4B 17 patients (2%). Class 5 has 316 patients (36.2%) and was further divided into 5.1 with a total of 153 patients (17.5%) and 5.2 with a total of 163 patients (18.7%). Class 6 has 41 patients (4.7%). Class 7 has 76 patients (8.7%). Class 8 has 27 patients (3.1%). Class 9 has 5 patients (0.6%). Finally, Class 10 has 69 patients (7.9%). 

## Discussion

Principal findings

Cesarean section procedure has been performed in the presentation of medical and non-medical indications concerning the mother or the infant [1]. In order to fulfil the aim of the study of assessing the rate of CS deliveries along with identifying the most contributing Robson groups to CS rate at KAMC in Jeddah, Saudi Arabia, Robson Classification System was used to classify the deliveries of 2018 at KAMC into 10 classes (Table 2). Statistical analysis showed that Class 5 (multiparous women with one or more previous uterine scar(s) and a single cephalic pregnancy ≥ 37 weeks of gestation) which is divided into Class 5.1 (17.5%) and Class 5.2 (18.7%), represents (36.2%) of all CS deliveries, making it the first-highest class contributing to the CS rate. Similar studies to ours was published during the last decade showed the same results. A study conducted in the UAE during 2017 concluded that the highest contributing group to their overall CS rate was group 5 which is the same as our study [7]. Another study was conducted in Egypt stating that Class 5 has the highest contributing percentage [8]. Lastly, a published Palestinian study stated that their highest contributing group was 5 as well [9]. Also, Class 5 was the major contributor to the CS rates in USA, France, Canada, Turkey, Lithuania, Tanzania, Ethiopia, and South Africa [9,12-13]. Therefore, great efforts are needed to improve labor management in women in this large contributing group in order to reduce CS rates worldwide. Following Class 5, Class 2 (primiparous women with a single cephalic pregnancy, ≥ 37 weeks of gestation, submitted to induction of labor or to CS prior to the onset of labor) is the second-highest contributing group (13%), and this class was further divided into two classes (2A and 2B) according to labor induction. Class 2A (women who had their labor induced) includes almost all the number of women in Class 2 while Class 2B has 12 women only. The third highest class in number of patients is Class 3, representing 9.2% of CS rate (multiparous, no previous CS, single cephalic pregnancy, term, spontaneous labor). However, the second and third highest contributing classes in the other studies differ. In high income countries, Class 2 was the second-highest contributing group while Class 1 was the third-highest contributing group [9]. However, in low-income countries where CS rates extremely low, Class 3 was the second-highest contributing group and Class 5 was the third-highest contributing group [9]. Following the three major contributing classes, comes Class 7, Class 1, Class 10, Class 4, Class 6, Class 8, and Class 9 in order of highest to lowest number of patients contributing to the overall CS rate. The WHO has expected that classes 1, 2, and 5 contribute to make up two thirds of the overall CS rates, however, in this study classes 1, 2, and 5 have already made up more than two thirds of the of the overall CS rate at KAMC, Jeddah. Although Class 1 and Class 2 are considered as uncomplicated pregnancies allowing them to deliver through vaginal delivery, both of their contributions make up more than two thirds of the overall CS rate. Also, according to the five main obstetric characteristics of the Robson Classification System, the high-risk groups that have greater indication for cesarean section procedure include the least number of women in comparison to the low-risk groups in this study. However, classes from 1 to 4 do not require CS procedures because they are considered as low-risk groups, indicating that KAMC has a high cesarean section delivery rate in low-risk patients. Even though, the low-risk group is generally the group that all hospitals usually target to reduce their CS rate. Thus, performing spontaneous vaginal delivery (SVD) for these patients instead of CS, if possible, will lower the rate of unnecessary CS deliveries that can lead to serious complications in both women and infants. The average global CS rate shows that Asia has an average CS rate of 19.2%, while the overall CS rate at KAMC, Jeddah is 27.5% making it much higher than the average rate of Asia. As reported by the WHO data in 2012, the CS rate in both Bosnia and Herzegovina is 15% which is the lowest rate recorded. In contrast, the highest rate of CS was recorded in Cyprus (50.9%) and Brazil (52.3%) [14]. Furthermore, the CS rate in Saudi Arabia accounts for 10% in all health centers, while the CS rate in tertiary hospitals is 20% [15]. Moreover, in King Fahd Armed Forces Hospital, Riyadh the CS rate has exceeded 20% in 2007 [15]. At KAMC, Riyadh, the overall CS rate has increased significantly from 8% to 27% between the years 1993 and 2016 [16]. On the other hand, the statistical analysis of this study showed that the overall CS rate at KAMC, Jeddah in 2018 was 27.5% of all total deliveries, and that shows that the CS rate at KAMC, Jeddah is higher than the CS rate of the tertiary hospitals in Saudi Arabia. Also, the CS rate of KAMC in Jeddah is higher than CS rates of both KAMC and in King Fahd Armed Forces Hospital in Riyadh. Therefore, the results highlight the importance of more evaluation and improvement of the interventions, management, and care provided to women in labor to reduce the CS rate. In a study that was conducted at KAMC, Riyadh in 2018, it was found that performing CS without medical indications, the willingness to undergo another CS in future deliveries without medical indications, low education level, having no personal vaginal delivery history, and older maternal age were the major contributing factors to the increase of CS rate from 20.3% to 27% between the years 2010 and 2016 [2]. Hence, the need of more education for both pregnant women and their physicians is required to promote vaginal deliveries and decrease the CS rate by preventing women from undergoing unnecessary CS. Our recommendation for a future study to be done in the same year and same hospital on patients who went to CS with Robson Class 1 to Class 4 to observe and further study the reasons for CS and the fetal outcomes for a possible conclusion on whether these patients could have delivered vaginally or not. As this is the first study that uses the Robson classification in the Kingdom of Saudi Arabia, we also recommend that similar studies to be done in other city health centers in the country so that in the future it is possible to do a multicenter systematic review in our chosen population. Finally, during the data collection phase a few limitations were faced namely the unavailability of approximately 30 medical records in the medical record department due to unexplained reasons, the time frame that was chosen was limited, and the assessment of other hospitals or centers was not feasible; however, these limitations could be looked into in future studies.

**Table 2:** The Robson TGCS. CS, cesarean section; TGCS, Ten Group Classification System

Group	Obstetric characteristics
1	Nulliparous women with a single cephalic pregnancy, ≥37 week gestation in spontaneous labor
2	Nulliparous women with a single cephalic pregnancy, ≥37 week gestation who had labor induced or were delivered by CS before labor
2A	Labor induced
2B	Pre-labor CS
3	Multiparous women without a previous CS, with a single cephalic pregnancy, ≥37 weeks gestation in spontaneous labor
4	Multiparous women without a previous CS, with a single cephalic pregnancy, ≥37 weeks gestation who had labor induced or were delivered by CS before labor
4A	Labor induced
4B	Pre-labor CS
5	All multiparous women with at least one previous CS, with a single cephalic pregnancy, ≥37 weeks gestation
5.1	With one previous CS
5.2	With two or more previous CSs
6	All nulliparous women with a single breech pregnancy
7	All multiparous women with a single breech pregnancy including women with previous CS(s)
8	All women with multiple pregnancies including women with previous CS(s)
9	All women with a single pregnancy with a transverse or oblique lie, including women with previous CS(s)
10	All women with a single cephalic pregnancy <37 weeks gestation, including women with previous CS(s)

Strengths of the study

The strengths of this study are that it may help obstetricians at KAMC, Jeddah to analyze and understand the causes of such a high CS rate, especially with how easily the TGCS is applicable. As well as practicing improved clinical judgement and helping educate mothers who choose CS birth over vaginal delivery electively.

Limitations of the study

During the data collection phase a few limitations were faced which were the unavailability of approximately 30 medical records in the medical record department due to unexplained reasons, the time frame that was chosen was limited, and the assessment of other hospitals or centers was not feasible; however, these limitations could be looked into in future studies.

## Conclusions

The Robson Classification System has proven to be feasible and useful to apply in all health institutions. Although a great deal of effort had been put into lowering the CS rate nationwide, the TGCS proves to be the most successful and cost-effective approach to lower the CS rate worldwide. While also preventing any medical practices to take place if nonevidence-based. Improved education of pregnant nulliparous women to prevent nonmedically indicated CS is needed in order to preclude repeated CS births in the future and further increase the CS rate.
